# New Allergens Approved by the WHO/IUIS Allergen Nomenclature Sub‐Committee in 2021–2024 and Their Significance for Future Diagnostics, Regulation, and Research. An EAACI Task Force Report

**DOI:** 10.1111/all.70166

**Published:** 2025-11-29

**Authors:** Christian Radauer, Gabriele Gadermaier, Richard E. Goodman, Alain Jacquet, Uta Jappe, Andreas L. Lopata, Anna Pomés, Monika Raulf, Keity S. Santos, Josefina Zakzuk, Yuzhu Zhang, Joana Vitte, the WHO/IUIS Allergen Nomenclature Sub‐Committee

**Affiliations:** ^1^ Institute of Pathophysiology and Allergy Research Center for Pathophysiology, Infectiology and Immunology, Medical University of Vienna Vienna Austria; ^2^ WHO/IUIS Allergen Nomenclature Sub‐Committee and EAACI Task Force on Allergen Nomenclature; ^3^ Department of Biosciences and Medical Biology University of Salzburg Salzburg Austria; ^4^ Food Allergy Research & Resource Program University of Nebraska—Lincoln Lincoln Nebraska USA; ^5^ Department of Biochemistry, Faculty of Medicine Chulalongkorn University Bangkok Thailand; ^6^ Division of Clinical & Molecular Allergology, Research Center Borstel, Leibniz Lung Center, Airway Research Center North (ARCN) German Center for Lung Research (DZL) Borstel Germany; ^7^ Department of Pneumology Interdisciplinary Allergy Outpatient Clinic, UKSH Campus Lübeck, University of Lübeck Lübeck Germany; ^8^ College of Science and Engineering Australian Institute of Tropical Health and Medicine, James Cook University Townsville Queensland Australia; ^9^ Tropical Futures Institute James Cook University Singapore Singapore, Singapore; ^10^ InBio Charlottesville Virginia USA; ^11^ Department of Allergology/Immunology, Institute of Prevention and Occupational Medicine of the German Social Accident Insurance (IPA) Institute of the Ruhr‐University Bochum Bochum Germany; ^12^ Departamento de Clínica Médica, Disciplina de Alergia e Imunologia Clínica Faculdade de Medicina da Universidade de São Paulo São Paulo Brazil; ^13^ Institute for Immunological Research University of Cartagena Cartagena de Indias Colombia; ^14^ US Department of Agriculture, Agricultural Research Service, Pacific West Area Western Regional Research Center Albany California USA; ^15^ Immunology Laboratory, Faculty of Medicine INSERM UMR‐S 1250 P3CELL, University Hospital of Reims, University of Reims Champagne‐Ardenne Reims France

**Keywords:** allergen nomenclature, allergens, climate change, genomics, globalization

## Abstract

The WHO/IUIS Allergen Nomenclature Sub‐Committee is an international body of experts that maintains the systematic nomenclature of allergenic proteins by assigning official names to newly identified allergens submitted by researchers. Here, we summarize the data on new allergens approved between 2021 and 2024. The sub‐committee assigned names to 112 new allergens with 124 isoallergens/variants as well as 26 new isoallergens/variants of previously named allergens. Most new allergens were respiratory allergens from animals (35 allergens) and plants (25) as well as food allergens from animals (22) and plants (17). Many newly identified allergens reflect globalized allergen exposure and growing research activities outside of Western countries. This is illustrated by allergens from the tropical mite *Blomia tropicalis*, pollen allergens from tree and weed species native to Asia, and food allergens from regionally important foods such as mango, seafood, silkworm pupae, and natto. The allergen profiles of most relevant sources are well established, but gaps in our knowledge remain, particularly regarding allergens important for populations outside of Europe and North America. The still growing number of known allergens highlights the importance of a consistent, unambiguous allergen nomenclature that evolves with clinical demands and scientific discovery and supports efforts to close existing knowledge gaps.

## Graphical abstract

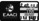

## 1 Introduction

The WHO/IUIS Allergen Nomenclature Sub‐Committee is an international body of experts in molecular allergy research responsible for maintaining and developing the systematic nomenclature for allergenic proteins. The allergen nomenclature was published first in 1986 [1] and revised in 1994 [2]. Adherence to this nomenclature is required by all peer‐reviewed scientific journals in the field of allergy and immunology. The allergen nomenclature is also used by clinicians, regulatory bodies and manufacturers to unambiguously identify allergens in diagnostic tests and therapeutic products. Data on approved allergen names are made available in the WHO/IUIS Allergen Nomenclature Database at https://www.allergen.org.

Researchers who identify a new candidate allergen are asked to submit its biochemical and clinical properties to the WHO/IUIS Allergen Nomenclature Sub‐Committee for review before publication of their data. Allergens will be approved if the submitted information meets the following criteria, as published previously [3] and outlined on the Allergen Submission page of the WHO/IUIS Allergen Nomenclature Database (https://www.allergen.org/submission.php):

- IgE binding data must be obtained using sufficiently purified natural or recombinant allergens.
- The expression of the allergen in a tissue or organ relevant for human exposure must be demonstrated at the mRNA or protein level.
- The submission must include the (at least partial) sequence of the allergen.
- IgE binding must be tested with sera from a sufficiently high number of patients allergic to the source of the allergen as diagnosed using common clinical standards.
- The proposed allergen should bind IgE from at least five of the tested sera.

Even 35 years after the molecular cloning of the first allergens, new allergens from previously known and novel sources are being identified. In this review article, we summarize data on new allergens approved by the WHO/IUIS Allergen Nomenclature Sub‐Committee between 2021 and 2024 and discuss specific developments in molecular allergy research.

## 2 Overview of New Allergens Approved in 2021–2024

Newly approved allergens are summarized in Table 1. Detailed lists containing all new allergens as well as new isoallergens and variants of previously approved allergens accompanied by associated references can be found in Tables S1 and S2 in the Supporting Information.

**TABLE 1:** Statistics of allergens newly approved between 2021 and 2024.

	Number of sources	Number of allergens	Number of isoallergens and variants
New allergens	47	112	124
New isoallergens/variants of previously approved allergens	10	13	26
New allergens classified by group of allergen sources			
Plant respiratory	12	25	25
Pollen	11	22	22
Other	1	3	3
Plant foods	10	17	27
Tree nuts, seeds	6	11	20
Fruits, tubers	4	6	7
Animal respiratory	7	35	36
Mites	5	25	25
Cockroaches	1	9	10
Mammals	1	1	1
Animal foods	13	22	23
Crustaceans, mollusks, insects	10	18	19
Fish	3	4	4
Animal venoms	3	5	5
Molds	1	7	7
Bacterial food	1	1	1

**FIGURE 1:**
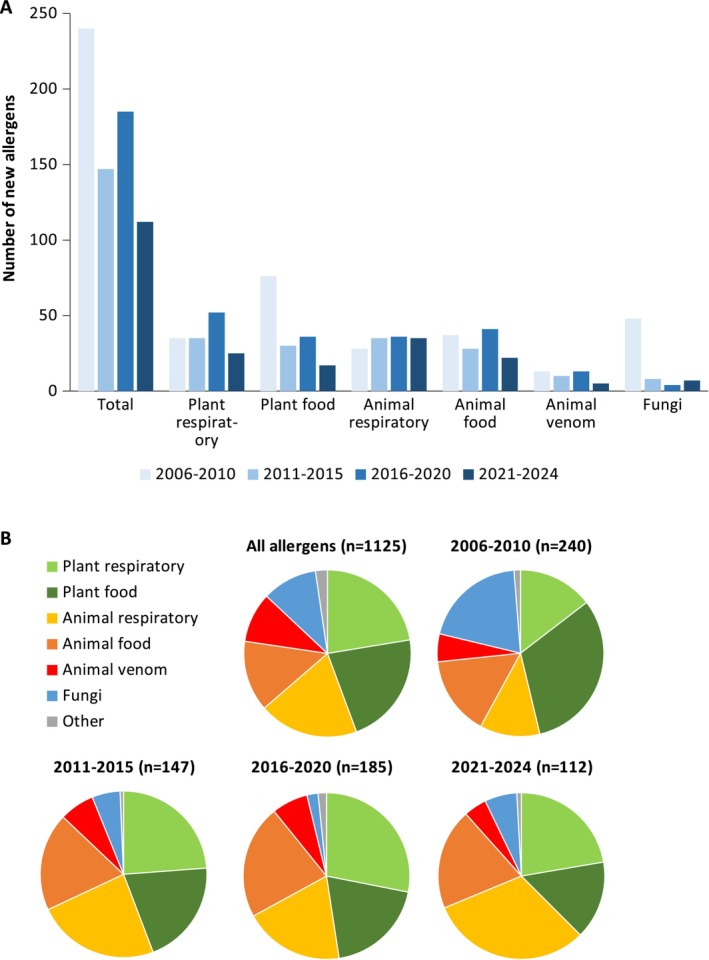
Development of allergen submission to the WHO/IUIS Allergen Nomenclature Sub‐Committee since 2006 grouped by type of allergen source as absolute values (A) and proportions (B).

Between 2021 and 2024, 112 allergens from 47 sources with 124 isoallergens and variants were assigned official allergen names. In addition, 26 new isoallergens and variants of 13 previously approved allergens were added. The species with the highest numbers of new allergens were the tropical dust mite *Blomia tropicalis* with 12 new allergens, the American cockroach ( *Periplaneta americana* ; 9 allergens), and the mold *Aspergillus fumigatus* (7 allergens). The groups of sources with the highest numbers of new allergens were respiratory allergens from animals (35 allergens) and plants (25) as well as food allergens from animals (22) and plants (17). Over the past 20 years, we have observed a slight decline in the numbers of new allergens in all groups of sources except respiratory allergens from animals (Figure 1A). Consequently, between 2021 and 2024, as many as 32% of all newly approved allergens fell into the latter category, comprising mostly allergens from mites and cockroaches (Figure 1B). These trends probably reflect the fact that most allergens from important sources have already been identified, whereas many new mite and cockroach allergens of unclear significance were found in omics‐based projects (see the section “Genomics and transcriptomics as tools to identify allergen candidates”).

## 3 Spotlights on Trends in Molecular Allergy Research

### 3.1 New Sources With Identified Allergens

Between 2021 and 2024, 24 allergens from 15 novel sources were added to the WHO/IUIS Allergen Nomenclature Database (Table 2). These sources comprise plant, animal, and bacterial species.

**TABLE 2:** Approved allergens from new sources.

Source	Allergen	Protein name
*Pollen*		
Paper mulberry ( *Broussonetia papyrifera* )	Bro p 3	Non‐specific lipid transfer protein class 1 (nsLTP1)
Sawtooth oak ( *Quercus acutissima* )	Que ac 1 Que ac 2	Bet v 1‐related protein, PR‐10 Profilin
*Plant food*		
Mango ( *Mangifera indica* )	Man i 1 Man i 2 Man i 4	Class IV chitinase Bet v 1‐related protein Profilin
Korean ginseng ( *Panax ginseng* )	Pana g 1	Bet v 1‐related allergen, PR‐10
Opium poppy ( *Papaver somniferum* )	Pap s 1 Pap s 2 Pap s 3	7S Globulin 11S Globulin Late embryogenesis abundant protein 5
Sichuan pepper (*Zanthoxylum bungeanum*)	Zan b 1 Zan b 2	2S Albumin 11S Globulin
*Animal food*		
Warrior swimming brown crab ( *Callinectes bellicosus* )	Cal b 2	Arginine kinase
Portuguese oyster ( *Crassostrea angulata* )	Cra a 1 Cra a 2 Cra a 4	Tropomyosin Arginine kinase Sarcoplasmic Ca‐binding protein
Spotted seabass (*Lateolabrax maculatus*)	Late m 1	Parvalbumin
Red king crab ( *Paralithodes camtschaticus* )	Para c 11	Mitochondrial malate dehydrogenase
Gazami crab (*Portunus trituberculatus*)	Por t 4	Sarcoplasmic Ca‐binding protein
Sole ( *Solea solea* )	Sole s 1	Parvalbumin
Atlantic cutlass ( *Trichiurus lepturus* )	Tric l 1	Parvalbumin
*Venom*		
Central American paper wasp (*Apoica pallens*)	Apo p 1 Apo p 5	Phospholipase A1 Antigen 5
*Bacterial food*		
Natto bacillus (*Bacillus subtilis subsp. natto*)	Bac s 1	Nattokinase (subtilisin‐like serine protease)

#### 3.1.1 Pollen Allergens

Bro p 3, first described in Pakistan and China [4], is the first allergen identified from pollen of the paper mulberry ( *Broussonetia papyrifera* ) tree, a significant source of aeroallergens in those countries. Paper mulberry is a widely distributed tree native to Eastern and Southeastern Asia and is cultivated or grows as an introduced species elsewhere [5]. Bro p 3 is a class 1 non‐specific lipid transfer protein (nsLTP1), belonging to a family of clinically relevant pollen and plant food allergens known for their heat stability and resistance to proteolysis [6]. While nsLTPs are major allergens in the Mediterranean region and in China [7], often causing severe reactions, their role elsewhere is less clear [8].

Sawtooth oak ( *Quercus acutissima* ) is a major cause of tree pollen allergy in Korea. It is native to Asia, but has become naturalized in parts of North America, and is widely planted elsewhere. Its pollen exhibits cross‐reactivity with other tree pollens, including white oak and birch. Two allergens were described: Que ac 1, a Bet v 1‐related protein, and Que ac 2, a profilin, which share homology with respiratory and plant food allergens [9]. Sensitization to Bet v 1 homologs is associated with clinically relevant reactions to tree pollen and plant foods such as apple, hazelnut, and peach [10]. In contrast, profilins exhibit broader IgE cross‐reactivity, though often with limited clinical relevance [11].

#### 3.1.2 Plant Food Allergens

Four new sources of plant food allergens were identified (Table 2). Most newly named allergens are members of well‐known protein families with potential cross‐reactivity to pollen and other plant foods.

Central and Eastern Europe, the Middle East and India have a tradition of growing culinary poppy ( *Papaver somniferum* ), and its seeds are often used in bakery products [12]. Although allergic reactions to poppy have previously been described [13, 14, 15], four allergens were identified only recently [16]. The 7S and 11S globulins (Pap s 1 and Pap s 2) cross‐reacted with hazelnut ( *Corylus avellana* ) and buckwheat (*Fagopyrum* spp.) homologs.

IgE‐mediated mango ( *Mangifera indica* ) allergy is the leading cause of fruit allergy in China and is associated with pollinosis to *Artemisia* and *Betula* pollens [17, 18]. Three allergens were identified (Table 2). A profilin, Man i 4, cross‐reacted with Art an 4 from *Artemisia annua* and may contribute to the association of *Artemisia* pollinosis with mango allergy [18].

The medicinal properties of Korean ginseng ( *Panax ginseng* ) are well known, but this plant can also induce allergies [19, 20]. A potentially cross‐reactive Bet v 1‐related allergen was named Pana g 1 (unpublished data submitted to the WHO/IUIS Allergen Nomenclature Sub‐Committee).

Sichuan pepper (*Zanthoxylum bungeanum*), a spice used worldwide although mostly grown in China, is also used as a traditional herbal medicine [21]. Zan b 1, a 2S albumin, and Zan b 2, an 11S globulin, cross‐reacted with extracts of cashew nuts, pistachios, and citrus seeds [21, 22].

These new allergens extend the range of identified allergens to previously underexplored foods. However, the clinical relevance of their cross‐reactivity with allergens from established pollen or food allergen sources at the population level remains to be determined.

#### 3.1.3 Animal Food Allergens

New food allergen sources of animal origin comprise seven species of seafood (Table 2). Invertebrate allergens from the arginine kinase protein family were identified in the swimming brown crab ( *Callinectes bellicosus* ; Cal b 2) in Mexico [23] and in the Portuguese oyster ( *Crassostrea angulata* ; Cra a 2) in China, which was cross‐reactive with crustacean homologs [24]. Additional allergens from the Portuguese oyster are the tropomyosin Cra a 1 [25] and the sarcoplasmic Ca‐binding protein Cra a 4 [26]. Two additional allergens from novel crab species are the Gazami crab (*Portunus trituberculatus*) sarcoplasmic Ca‐binding protein, Por t 4 [27], and the red king crab ( *Paralithodes camtschaticus* ) mitochondrial malate dehydrogenase, Para c 11 [28]. Finally, three fish species, spotted seabass (*Lateolabrax maculatus*) [29], sole ( *Solea solea* ), and Atlantic cutlass ( *Trichiurus lepturus* ), are new sources of allergens from the parvalbumin protein family.

#### 3.1.4 Venom Allergens

Two novel allergens from the venom of the Central American paper wasp (*Apoica pallens*), Apo p 1 and Apo p 5, were identified [30]. *Apoica pallens* is a nocturnal social wasp from the family Vespidae distributed across Central and South America [31]. Apo p 1 and Apo p 5 belong to the phospholipase A1 and antigen 5 protein families, respectively. Both protein families contain highly relevant and cross‐reactive major allergens identified in venoms from Vespid and Hymenoptera species, respectively [32, 33].

#### 3.1.5 Bacterial Food Allergens

The first allergen of bacterial origin listed in the WHO/IUIS database is nattokinase (Bac s 1) from *Bacillus subtilis subsp. natto*. Bac s 1 is a subtilisin‐like serine protease responsible for allergic reactions to the traditional Japanese fermented soybean dish natto [34]. Proteins of *Staphylococcus aureus* have been identified as IgE‐binding superantigens but also as allergens [35, 36, 37], but have not been submitted to the WHO/IUIS Allergen Nomenclature Sub‐Committee.

These registered allergens from new sources will contribute to allergy diagnosis in specific areas of the world where these species live. Even if uncommon, these allergens might be an important cause of cross‐reactivity with homologous allergens. Analyses of IgE sensitization patterns might eventually improve patient care in specific regions.

### 3.2 Globalization, Climate Change and Their Impact on the Allergen Exposome

**FIGURE 2:**
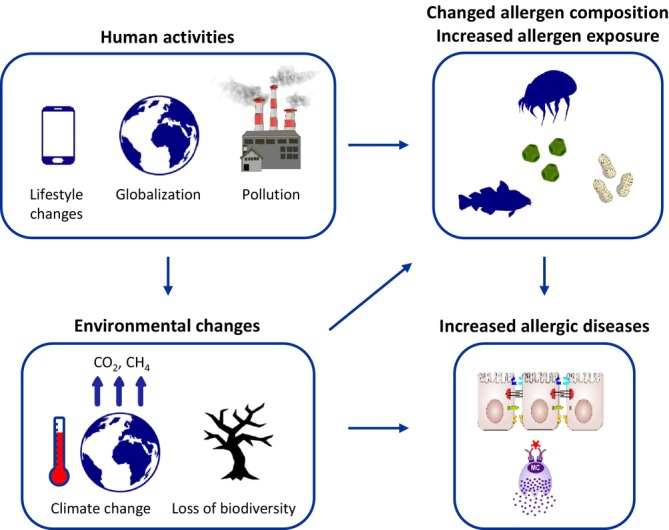
Human activity associated with pollution, loss of biodiversity, globalization and lifestyle changes affects climate, allergen sources and human health. The nature, quantity and potency of allergens change as a result of anthropogenic climate change, lifestyle, and occupation. Epithelial barriers and immune responses are impaired by pollution, climate stress, loss of biodiversity, lifestyle changes, and occupational exposures. Interactions between leaky epithelial barriers and novel allergens manifest as allergy to novel sources or novel allergens.

A variety of factors contributing to the increased incidence of allergic diseases in the past century has been discussed [38]. These include changes in the microbiota and disruption of epithelial barriers due to excessive hygiene and contact with irritant compounds [39], changes in children's lifestyle with lower physical activity and more time spent indoors, and increased exposure to certain allergen sources, both outdoors and indoors. The risk imposed by new or increased allergen exposure is illustrated by respiratory allergies linked to increasing airborne pollen and fungal spore counts [40, 41, 42] and emerging allergies to novel foods [43]. These environmental and lifestyle changes contribute to asthma and allergic diseases (Figure 2) [44, 45]. Allergens added to the WHO/IUIS Allergen Nomenclature Database between 2021 and 2024 (Table S1) may reflect the direct or indirect association of climate change and globalization with allergic diseases.

#### 3.2.1 Climate Change

Rising temperatures, more frequent extreme weather events, and shifts in precipitation patterns influence outdoor and indoor environmental exposure by altering natural habitats of plant, fungal, and animal species, pollen seasonality, concentrations, and allergenicity [46], as well as reproduction of fungi, mites and insects [42, 47, 48, 49]. Extreme weather events also disrupt infrastructure, often resulting in decreased hygiene, which favors fungal growth and pests such as mites and cockroaches [49].

The establishment and spread of exotic mosquito species, such as the Asian tiger mosquito ( *Aedes albopictus* ), and the yellow fever mosquito ( *Aedes aegypti* ), in Europe and the US have increased recently [50, 51]. Their bites can cause sensitization and cross‐reactivity with allergens from native species, for example via Aed al 13, a member of the antigen 5 protein family [52].

Cockroaches as a climate change‐related allergen source are represented by the American cockroach, which is the source of 20 currently identified allergens, nine of which have been characterized since 2021 [53, 54, 55]. Twenty‐five new allergens were characterized in mites, which thrive in damp and warm conditions. This includes 12 allergens from *B. tropicalis* , which is increasingly spreading to temperate areas [56].

Seven allergens were newly identified in *Aspergillus fumigatus* [57], one of the main fungal sensitizers, predominantly present indoors, and associated with lung function deterioration, severe asthma, allergic fungal rhinosinusitis, and allergic bronchopulmonary aspergillosis [42, 57].

#### 3.2.2 Global Food Trends

Globalization and the trend toward sustainable nutrition foster the introduction of previously underexplored food sources, including algae, fungi, hemp, insects, and various legumes, the expansion of aquaculture [58] as well as food innovations including cell‐based meat and novel foods produced by bacterial fermentation.

Food globalization is probably best reflected by the high number of newly identified fish and shellfish allergens (22 allergens from 13 species; Table 1, Table S1). Insects have been gaining momentum in Western menus as a climate‐friendly and cost‐effective source of proteins, unsaturated fatty acids, and other nutrients. Four new allergens were characterized from silkworm ( *Bombyx mori* ) [59, 60, 61], one of the most widely used species for both human food and animal feed [62].

Relating to the “healthy food” trend, allergens were characterized in Korean ginseng, mango [18], hazelnut [63], macadamia nut ( *Macadamia integrifolia* ) [64], buckwheat [65], and sunflower ( *Helianthus annuus* ) seeds [66]. Another example is the bacterial allergen Bac s 1, a subtilisin from natto [34]. While natto is predominantly consumed in Japan, the potential health benefits of natto and nattokinase ingestion are an area of global research [67].

#### 3.2.3 Lifestyle Changes and Occupational Health

Hemp ( *Cannabis sativa* ) has experienced a resurgence as a bio‐based raw material, providing cellulose, textile fibers, and oil. It is also increasingly used in lifestyle products, raising employment in this sector. However, both recreational and occupational exposures to cannabis have been linked to health issues, including allergic reactions [68, 69]. The newly described allergens, Can s 2, Can s 5, and Can s 7, belong to the profilin, Bet v 1‐related, and thaumatin‐like families of allergens with potential cross‐reactivities with respiratory and ingested allergen sources [70, 71].

Taken together, these examples illustrate how newly registered allergens reflect changes in allergen exposure due to climate change and globalization. Newly identified allergens may become tools for detecting emerging allergy risks to inform intersectoral health strategies. These include mitigation strategies for indoor and urban spaces, updating public health policies, and patient education programs [72].

### 3.3 The Increase in Allergen Research in Asia

**FIGURE 3:**
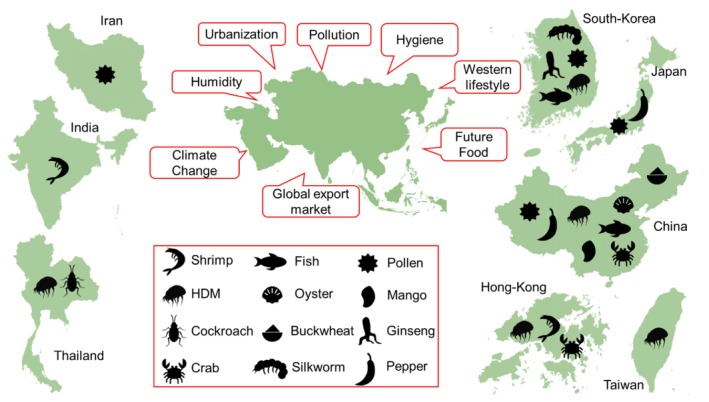
Multiple factors contribute to the rise of allergic disorders in Asia. New airborne and food allergens were identified from allergenic sources predominant in Asia.

Asia, with 4.8 billion people, is the most populated continent and has seen profound economic development, urbanization, improvement in hygiene and adoption of a lifestyle similar to that of the industrialized West. Environmental factors such as inner‐city pollution, extreme temperatures, and humidity have contributed to the increasing prevalence of allergic disorders across Asia in recent decades (Figure 3) [73, 74, 75, 76]. In this context, it is noteworthy that of 112 new allergens recently added to the database, more than 70% were identified in Asia. This highlights the ongoing advancement in allergy research within Asia, particularly in the more developed Asian countries.

Plant‐derived proteins are gaining worldwide popularity, with increased consumption of health‐promoting foods such as ginseng and buckwheat [77], both sources with newly identified allergens [65]. One of the drivers behind the identification of emerging allergens is the global trend toward alternative protein sources, with an expected increase in alternative meat and seafood consumption [78]. This includes the growing consumption of insects, not only in Asia but increasingly in Western countries as well, highlighted by the allergens recently identified in silkworm pupae [59, 60, 61], which have been consumed in most Asian countries for over a millennium [79]. Sources of new seafood allergens include mostly crab species such as the crucifix crab (*Charybdis feriata*), which is of cultural significance in many Asian countries and supports a growing commercial fishery. However, globally the Gazami crab is the most captured crab species with over 400,000 tons in 2021 [58]. The first Gazami crab allergen (Por t 4) was characterized by a research team from China [27]. It is anticipated that, with the increasing population in Asia and consumption of this species, allergic reactions to Gazami crab will be reported more frequently.

Mites and cockroaches are key indoor allergen sources in Asia, with sensitization rates rising probably due to Western lifestyle—smaller, air‐conditioned spaces increase exposure [80]. Common allergenic mite species include *Dermatophagoides pteronyssinus* and *D. farinae*, while *B. tropicalis* and *Tyrophagus putrescentiae* also cause significant allergic symptoms, especially in Southeast Asia, Southern China, and South Korea. Collaborative research in Hong Kong and Thailand described 12 new allergen groups from *B. tropicalis* [81, 82]. Thirteen new allergens were identified in other mite species in China and South Korea [83, 84, 85, 86, 87, 88]. Of note, Der f 42, a Na/K‐ATPase is the first identified membrane‐associated HDM allergen [88]. Moreover, researchers from Thailand, Singapore and China identified eight new American cockroach allergens [53, 54].

Rising pollen counts in Asia, linked to global warming, have contributed to increased incidences of allergic disorders [73]. Asia is warming faster than the global average, with frequent heatwaves [89]. Chinese researchers identified new pollen allergens from the plane tree ( *Platanus acerifolia* ) [90, 91, 92], giant ragweed ( *Ambrosia trifida* ) [93], *Artemisia* species [94, 95, 96], and paper mulberry [4]. *Artemisia* exposure has risen due to uncontrolled growth and warming, while species distribution models predict expanded spread of *Ambrosia* in the coming decades [97]. In South Korea and Japan, allergens from Japanese hop ( *Humulus japonicus* ) [98], sawtooth oak [9], and Japanese cedar ( *Cryptomeria japonica* ) [99] pollen were also submitted, approved and named, reflecting the impact of regional vegetation on allergic disease trends.

### 3.4 Genomics and Transcriptomics as Tools to Identify Allergen Candidates

**FIGURE 4:**
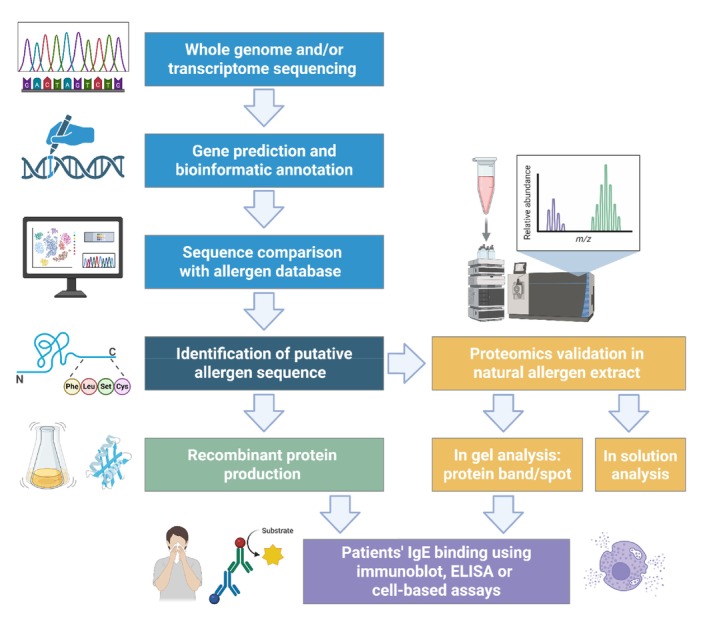
Strategy for the identification and characterization of novel allergens using genomics and transcriptomics information. The process integrates bioinformatic prediction, molecular biology techniques, and immunological assays to evaluate the allergenic potential. Created in https://BioRender.com.

Technological advances in omics sciences, particularly genome characterization, either by isolated genome assembly or multi‐omics approaches combining genomics with transcriptomics, proteomics, or both, have significantly accelerated allergen discovery and contributed to the expansion of the WHO/IUIS Allergen Nomenclature Database. Various approaches have been employed, including gene and transcript prediction, analysis of transcript sequences, and comparisons with allergen sequence databases such as the WHO/IUIS Allergen Nomenclature Database, AllergenOnline [100] and others (Figure 4). These strategies led to the identification of many potential allergens, some of which were expressed as recombinant proteins for further evaluation of their allergenicity.

While genomics approaches have significantly advanced the identification of putative allergens, some limitations should be considered. These methods alone do not provide information on protein expression levels, tissue specificity, or environmental relevance—factors that are important for determining the allergenic potential. Furthermore, genomic prediction of allergenicity cannot replace analysis of IgE reactivity and does not account for post‐translational modifications, which can influence allergenicity. Overprediction of allergenicity is a concern, as sequence homology of a protein to an allergen does not always translate into its clinical relevance [101]. For example, six potential *B. tropicalis* allergens were predicted based on sequence homology. While four candidates were confirmed to bind IgE from mite‐allergic patients, homologs of Der f 25 and Der f 31 showed only low IgE reactivity and therefore did not qualify as allergens [82]. Incomplete gene annotation and inconsistencies across allergen databases may further complicate allergen classification.

To address these limitations, integrating proteomic data into the allergen discovery workflow enables a more comprehensive view of allergen profiles and improves specificity in allergen identification as only expressed allergens are considered [102]. Omics‐driven allergen discovery can improve diagnostics, as demonstrated by the identification of Api g 7, a clinically relevant celery allergen, which is underrepresented in celery extract and was only identified using genomics and proteomics [103].

The following paragraphs summarize studies that used omics to identify novel allergens that were added to the WHO/IUIS Allergen Nomenclature database between 2021 and 2024 (Table 3).

**TABLE 3:** Genomes and proteomes of allergen sources used for allergen identification between 2021 and 2024.

Species	Genomics	Transcriptomics	Proteomics	New allergens	References
*Blomia tropicalis*	1601 contigs of 61 Mb in length	Yes 18,164 predicted genes	Yes 3714 proteins	None	[102]
	63.7 Mb; 14,899 protein‐coding genes, 183 tRNA genes and 40 rRNA genes.	Yes	No	Blo t 18 Blo t 24 Blo t 26 Blo t 41	[82]
*Tyrophagus putrescentiae*	18,763 predicted protein‐encoding genes	Yes	No	Tyr p 11 Tyr p 20	[87]
*Periplaneta americana*	3.06 Gb; 29,939 predicted protein‐coding genes	Yes; 182 potential allergenic hits, filtered based on identity, expression level and presence of potential epitopes showed 135 hits, 112 new sequences evaluated for allergenic potential	Yes; 2D IgE immunoblot and proteomics	Per a 7.02 Per a 14 Per a 15 Per a 16 Per a 17 Per a 18 Per a 19 Per a 20	[54]
*Penaeus monodon*	No	NCBI and UniProt (including [104])	Yes, IgE immunoblot and proteomics	Pen m 14	[105]

#### 3.4.1 Mites

Among the sequenced allergenic mite genomes are *B. tropicalis* , *D. pteronyssinus*, *D. farinae*, and *T. putrescentiae*. Although other mite species with allergenic potential have been sequenced, their data have not yet been integrated into allergenicity prediction workflows [101].

Through genome‐wide prediction, Xiong et al. identified 37 putative allergen groups in *B. tropicalis* (including previously reported allergens) and produced six putative allergens as recombinant proteins [82]. Three of these (Blo t 18, Blo t 26, and Blo t 41) showed significantly elevated reactivities with IgE from mite‐allergic patients (Table 3). Blo t 24, despite low IgE reactivity, was also accepted as an allergen by the WHO/IUIS Allergen Nomenclature Sub‐Committee. Hubert et al. conducted a multi‐omics study of *B. tropicalis* that combined genomics, transcriptomics, and proteomics [102]. This project has not yet submitted candidate allergens to the WHO/IUIS Allergen Nomenclature Database. However, its data enabled the identification of new isoforms and revealed that there is no coding sequence for “Blo t 19”, suggesting that this is not a true mite allergen but rather a nematode‐derived culture contaminant. Proteomic analyses complemented knowledge on allergens newly identified by Xiong et al. [101] For instance, Blo t 24, a ubiquinol‐cytochrome c reductase binding protein, was found exclusively in mite bodies and exhibited low abundance. In contrast, Blo t 18, a chitinase recognized by 73% of tested sera, was identified as the fifth most abundant protein in *B. tropicalis* extract [102].

Before the genome characterization of *T. putrescentiae*, 12 allergens had been officially identified. From the list of putative allergens predicted from the genome (> 20 allergens), two were produced as recombinant proteins and tested for their allergenic activity (Table 3). Tyr p 11 and Tyr p 20 are a paramyosin and an arginine kinase recognized by 73% and 45% of asthmatic individuals, respectively. The abundance of these proteins in natural mite extracts, however, was not investigated [87].

#### 3.4.2 Cockroaches

Genomics, transcriptomics and proteomics data of German cockroach ( *Blattella germanica* ) [106, 107] are available in public databases, but allergenicity was not investigated. Recently, genomic analyses of the American cockroach were conducted (Table 3). Predictions of putative allergens considering sequence identity, mRNA expression levels as well as T‐ and B‐cell epitopes revealed 23 previously identified and 112 novel predicted allergens with high sequence similarity with other cockroach, mosquito, and mite allergens [54]. To verify IgE binding, cockroach extract was analyzed by 2D gel electrophoresis, IgE‐immunoblotting and mass spectrometry. Seven new allergens (Per a 14–Per a 20) and Per a 7.02, a novel isoallergen of tropomyosin, were included in the WHO/IUIS Allergen Nomenclature Database. IgE ELISA with the recombinant allergens revealed greater than 65% sensitization frequencies for all allergens except Per a 15 [54].

#### 3.4.3 Shrimp

Genome assembly data of five *Penaeus* species are available in the NCBI database [108]. Comparison of the transcriptomes with allergen databases identified 39 potential novel allergens in those species [104]. IgE binding to black tiger shrimp ( *Penaeus monodon* ) was investigated using sera of shrimp‐allergic patients in Hong Kong and Thailand [105]. Proteomic analyses revealed four new IgE‐binding proteins (Table 3). A 95 kDa glycogenphosphorylase‐like protein was registered as Pen m 14. This allergen showed a high sensitization prevalence and, despite low amounts of specific IgE (sIgE), was suggested as a marker for shrimp allergy in individuals from Hong Kong. Enolase and aldolase showed low sensitization rates, attributed to the fact that those proteins are heat‐labile and food exposure typically occurs to heat‐treated shrimp [105]. Additional candidates obtained from transcriptome analyses were suggested as allergens [104]. However, IgE‐binding remains to be determined.

In conclusion, these examples demonstrate that the accelerated rate of allergen discovery facilitated by omics technologies makes it even more important to rigorously test the clinical relevance of newly identified allergens.

### 3.5 Assessing the Clinical Relevance of Allergens

**FIGURE 5:**
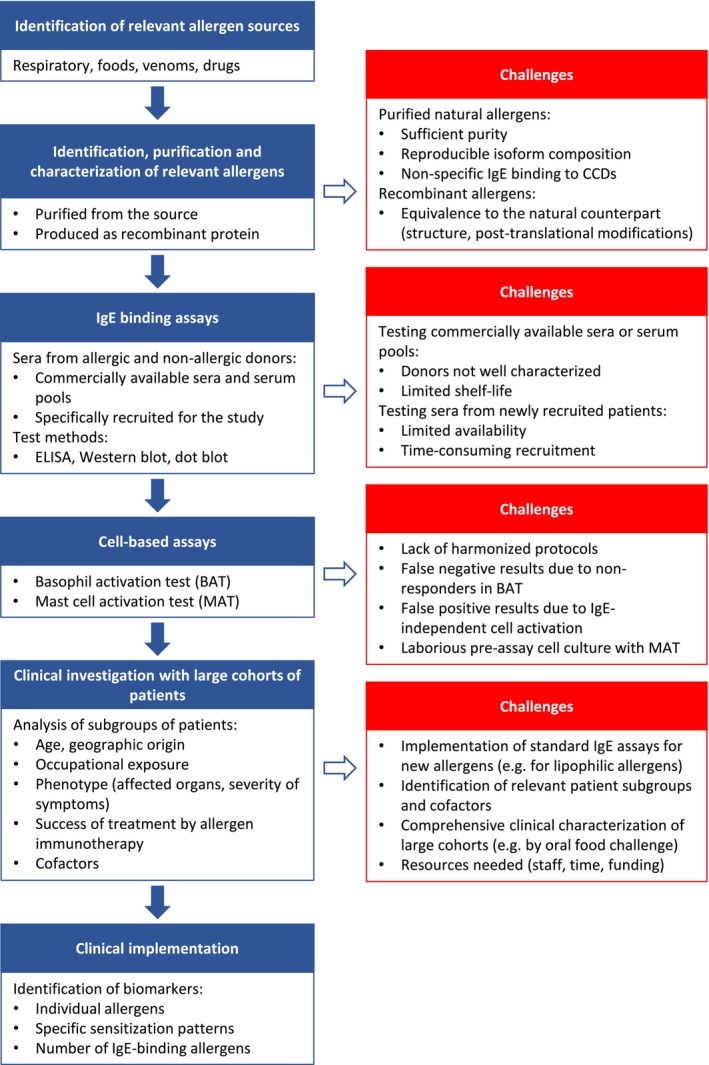
Workflow from allergen discovery to clinical implementation of new allergens and challenges encountered in this process.

Assessment of new allergen candidates for inclusion into the WHO/IUIS Allergen Nomenclature Database does not involve a judgment on their clinical significance. Allergen candidates are primarily evaluated for their capacity to bind IgE from patients who are genuinely allergic to the source of the allergen. Assessment of clinical relevance can only be achieved later with well‐characterized patient cohorts in a well‐equipped study setting, which requires appropriate resources, such as trained personnel and funding. However, such studies are possible only after newly documented allergens have become available for routine clinical testing (Figure 5).

Detection of sIgE to allergen sources and individual allergens as performed in clinical routine only documents an individual's sensitization but does not prove allergy. Instead, it is exactly the opposite. The clinical history is supported by the presence (or absence) of sIgE to suspected causative allergen sources (chapter A03 in Dramburg et al. [109]). However, a lack of IgE reactivity does not necessarily rule out a clinically established allergy. A false‐negative IgE test may have several reasons, such as a low abundance or even absence of the allergens in question in the diagnostic extract, as observed particularly with lipophilic allergens such as oleosins [110], or very low concentrations of sIgE. On the other hand, the lack of symptoms despite positive sIgE may be caused by asymptomatic sensitization or the dependence of allergic symptoms on cofactors, such as exercise, alcohol intake or medication [111, 112]. The gold standard of allergy diagnosis in difficult cases is still the allergen challenge test, which should include cofactors where applicable.

A lack of clinical relevance may be even more difficult to prove and requires long‐term observations of patients with different phenotypes. Some allergens may cause symptoms only in specific subgroups of patients or specific settings, such as occupational exposure [113]. Hence, they might be overlooked in research studies with small cohorts.

Some allergens have the potential to serve as markers for the existence and/or severity of an allergic reaction to a specific source. Many of these allergens belong to certain protein families. For instance, families of relevant plant food allergens include storage proteins (2S albumins, 7S globulins and 11S globulins), oleosins, as recently documented for buckwheat [65], defensins, newly documented for Api g 7 from celery ( *Apium graveolens* ) [114], and gibberellin‐regulated proteins [115] as identified recently in bell pepper ( *Capsicum annuum* ) [116]. Moreover, nsLTPs are relevant plant food and pollen allergens in certain sources, such as paper mulberry pollen, where Bro p 3 was described as a major allergen [4]. Other protein families contain marker allergens for respiratory allergen sources; for instance group 1 and 5 grass pollen allergens, group 1, 2 and 23 mite allergens, and Bet v 1 as a marker for allergy to pollen from birch and other Fagales trees [109].

As individual allergens are not available for challenge tests and only in experimental settings for skin tests, their actual clinical significance is hard to measure. Cell‐based assays may fill the gap, although they do not equally reflect clinical symptoms occurring under conditions of natural allergen exposure. Recently, however, a basophil activation test was optimized to allow for the differentiation between genuine allergy and asymptomatic sensitization [117]. This assay is presently being validated and tested with additional allergens. In addition, the passive mast cell activation test has been developed and will have to be validated for different allergens [118].

In summary, reliable tools do not exist so far to assess the clinical relevance of allergens submitted to the WHO/IUIS Allergen Nomenclature Sub‐Committee or of most of those that have already been named. In this regard, the WHO/IUIS Allergen Nomenclature Sub‐Committee and molecular allergologists in general still face a number of challenges:

- There is a lack of standard procedures for the evaluation of the clinical relevance of candidate allergens.
- Many of the individual allergens documented in the WHO/IUIS Allergen Nomenclature Database are not available for large studies with well‐characterized allergic patients. There is a lack of data regarding their relevance, sometimes even with regard to their IgE binding frequencies in well‐defined patient cohorts.
- The abundance of the allergens in the source materials and minimum symptom‐eliciting doses of the allergens and their source materials are not known. Marked differences can occur even between closely related species, as demonstrated for the three most important lupine species used for food production [119].
- Assays performed with recombinant allergens are potentially misleading. Depending on the expression system, the recombinant allergen may not be equivalent to its natural counterpart, concerning isoallergen composition, three‐dimensional structure and post‐translational modifications. Comparisons between naturally purified and recombinant counterparts are often lacking.
- Conversely, assays performed with purified natural allergens can yield false positive results, as even minute contaminations with other allergens or the presence of cross‐reactive carbohydrate determinants can affect the test results.

A related topic is assessing the risk of allergic reactions to foods derived from genetically modified organisms and other types of novel foods, as required by national and regional regulatory agencies. This procedure involves a sequence‐based comparison of novel food proteins with a database of established allergens. However, a report of the European Food Safety Agency concluded that none of the available *in silico* tools for allergenicity prediction was designed to account for clinical relevance in the evaluation of the search results [120]. In this context, it has to be stressed that the WHO/IUIS Allergen Nomenclature Database is not suitable to be used as a target database for allergenicity risk assessment.

## 4 Conclusions and Outlook

The WHO/IUIS Allergen Nomenclature Database provides the standard official nomenclature of allergenic proteins, which facilitates communication among researchers, clinicians and manufacturers of diagnostic and therapeutic allergen products. While the first published official list of allergen names contained only 56 allergens from 27 species [1], the number of named allergens has grown to now 1127 allergens from 322 species (as of June 2025) and is still growing.

As shown in this review article, several trends contribute to this growth: (1) Adoption of a Western lifestyle in many parts of the world increasing the incidence of allergic diseases has led to the emergence of novel environmental and food allergen sources native to regions outside Europe and North America and boosted research activities in those regions. (2) Globalization and climate change have brought about the deliberate or unintended introduction of potentially allergenic species into new regions, where they may sensitize atopic individuals or induce allergic reactions due to cross‐reactivity with local allergen sources. (3) New technologies such as high‐throughput omics approaches have facilitated the discovery of IgE‐binding proteins, which have resulted in the identification of multiple novel allergens in already well‐researched sources such as mites and cockroaches.

The WHO/IUIS Allergen Nomenclature Sub‐Committee continuously reviews submissions of new allergens and works on improving the database by adding missing previously identified allergens, deleting erroneous or obsolete entries, and optimizing the user interface. In addition, we invite the readers to report errors to the science co‐chair, whose contact details can be found at https://www.allergen.org.

## 5 Major Milestone Discoveries

- Major allergens were identified in sources of regional importance outside of Europe and North America, such as paper mulberry and sawtooth oak pollen, mango, Korean ginseng, Sichuan pepper, natto, and Central American Paper wasp.
- Asia has emerged as a leading region for the discovery of newly characterized respiratory and food allergens
- The naming of the first bacterial allergen, nattokinase from *Bacillus subtilis* ssp. *natto*, expanded the boundaries of known allergen sources from exclusively eukaryotic to include prokaryotes.
- Reflecting globalization of food supply, the spectrum of seafood species with identified allergens was complemented by various species of fish, crabs and oysters.
- Many newly identified allergens reflect the growing exposure to certain allergen sources due to global warming, including allergens from mosquitos, tropical mites, cockroaches, molds, and weed pollen.
- Genomics, transcriptomics and proteomics approaches fostered the discovery of many new allergens, particularly in mites and cockroaches.

## 6 Future Research Perspectives

- The current official list of named allergens is still biased toward allergens important for populations in Westernized countries. Future research should aim at complementing the database with allergens important in Asia, Africa and Latin America.
- The clinical relevance of many allergens listed in the WHO/IUIS Allergen Nomenclature Database is still to be established.
- Rigorous, standardized procedures need to be developed for testing allergen candidates derived from high‐throughput omics approaches, to prevent the inclusion of large numbers of clinically irrelevant allergens to the database.

## Author Contributions

All authors made substantial contributions to the conception and design of the article, were involved in drafting and revising the manuscript and gave final approval of the version to be published. C.R., G.G., A.J., U.J., A.L.L., A.P., K.S.S., J.Z., Y.Z. and J.V. prepared tables and figures.

## Conflicts of Interest

A.P. is an employee of InBio, and participation in this publication was in part supported by an R01 award of the National Institute of Allergy and Infectious Diseases of the National Institutes of Health. G.G. is a scientific expert of the European Food Safety Agency (EFSA) and a reviewer of the COMPARE database. U.J. has received two hotel accommodations and catering for a lecture and chairing of a workshop/symposium organized by ALK Abello. One respective honorarium went to her institution, the RCB. Her research on molecular allergology is funded by the Federal Ministry of Education and Science (BMBF) (DZL and INDICATE‐FH), the Federal Ministries of Technology, Economy and Technology, Food and Agriculture (BMEL), the German Research Foundation (DFG) (JA 1007/2‐1; JA 1007/2‐3; JA 1007/4‐1), and the Kanert Foundation. J.V. reports speaker and consultancy fees in the past 5 years from Astra Zeneca, HpVac, L'Oréal, Novartis, Sanofi, Thermo Fisher Scientific, Zambon, and travel expenses reimbursement from Stallergènes‐Greer for an international meeting, outside the submitted work. All other authors declare that they have no conflicts of interest in connection with this work.

## Supporting information


**Appendix S1:** all70166‐sup‐0001‐AppendixS1.pdf.

## Data Availability

Data sharing not applicable to this article as no datasets were generated or analyzed during the current study.
