# Supplementary material for: New Allergens Approved by the WHO/IUIS Allergen Nomenclature Sub‐Committee in 2021–2024 and Their Significance for Future Diagnostics, Regulation, and Research. An EAACI Task Force Report
Source: Allergy. 2025 Nov 29;81(3):684–99. doi: 10.1111/all.70166 (PMC12954567; doi:10.1111/all.70166)
Supplement: Supplementary file 1 — Appendix S1: all70166‐sup‐0001‐AppendixS1.pdf. [file ALL-81-684-s001.pdf]

Table S1. New allergens and their isoallergens/variants approved by the WHO/IUIS Allergen Nomenclature Sub-Committee between 2021 and 2024. Bold: new allergen sources.

Source	Allergen name	Isoallergens and variants	Biochemical names	Route of exposure	References
Aedes aegypti (Yellow fever mosquito)	Aed a 12	Aed a 12.01	Triosphosphate isomerase	Injection	-
Aedes albopictus (Asian tiger mosquito)	Aed al 13	Aed al 13.0101	Antigen 5-3	Injection	1
	Aed al 14	Aed al 14.0101	Salivary antigen LIPS-2 / 34k-2	Injection	1
Ambrosia trifida (Giant ragweed)	Amb t 13	Amb t 13.0101	Superoxide dismutase	Respiratory	-
	Amb t 18	Amb t 18.0101	Triosephosphate isomerase	Respiratory	2
Apium graveolens (Celery)	Api g 7	Api g 7.0101	Defensin-like protein 1	Food	3
***Apoica pallens* (Central American paper wasp)**	Apo p 1	Apo p 1.0101	Phospholipase A1	Venom	4
	Apo p 5	Apo p 5.0101	Antigen 5	Venom	4
Artemisia annua (Sweet Wormwood)	Art an 4	Art an 4.0101	Profilin	Respiratory	5
	Art an 14	Art an 14.0101	Fructose bisphosphate aldolase	Respiratory	6
Artemisia sieversiana (Sieversian wormwood)	Art si 5	Art si 5.0101<br>Art si 5.0201	Polcalcin	Respiratory	7
	Art si 6	Art si 6.0101	Pectate lyase	Respiratory	8
	Art si 8	Art si 8.0101	Superoxide dismutase	Respiratory	-
	Art si 12	Art si 12.0101	Enolase	Respiratory	-
	Art si 14	Art si 14.0101	Fructose bisphosphate aldolase	Respiratory	6

Source	Allergen name	Isoallergens and variants	Biochemical names	Route of exposure	References
Aspergillus fumigatus (Common mold)	Asp f 19	Asp f 19.0101	Heat shock protein 70 kDa (Hsp70)	Respiratory	9
	Asp f 24	Asp f 24.0101	Putative eukaryotic translation elongation factor 1 subunit EF1-beta	Respiratory	9
	Asp f 35	Asp f 35.0101	Cu-Zn Superoxide dismutase	Respiratory	9
	Asp f 36	Asp f 36.0101	Fructose-bisphosphate aldolase	Respiratory	9
	Asp f 37	Asp f 37.0101	NAD-dependent malate dehydrogenase	Respiratory	9
	Asp f 38	Asp f 38.0101	Protein of unknown function	Respiratory	9
	Asp f 39	Asp f 39.0101	FG-GAP repeat protein	Respiratory	9
***Bacillus subtilis ssp. natto* (Natto bacillus)**	Bac s 1	Bac s 1.0101	Nattokinase (subtilisin-like serine protease)	Food	10
Blomia tropicalis (Storage mite)	Blo t 9	Blo t 9.0101	Trypsin-like serine protease	Respiratory	-
	Blo t 16	Blo t 16.0101	Gelsolin/villin	Respiratory	-
	Blo t 18	Blo t 18.0101	Chitinase-like protein (contains a C-terminal peritrophin-A-like domain)	Respiratory	11
	Blo t 24	Blo t 24.0101	Ubiquinol-cytochrome c reductase binding protein; Cytochrome b-c1 complex subunit 7	Respiratory	-
	Blo t 26	Blo t 26.0101	Myosin light chain	Respiratory	11
	Blo t 27	Blo t 27.0101	Serpin serine protease inhibitor	Respiratory	-
	Blo t 28	Blo t 28.0101	Heat shock protein Hsp70	Respiratory	-
	Blo t 30	Blo t 30.0101	Ferritin	Respiratory	-
	Blo t 31	Blo t 31.0101	Cofilin	Respiratory	-

Source	Allergen name	Isoallergens and variants	Biochemical names	Route of exposure	References
	Blo t 32	Blo t 32.0101	Inorganic pyrophosphatase	Respiratory	-
	Blo t 37	Blo t 37.0101	Chitin binding protein (contains 2 peritrophin-A-like domains)	Respiratory	-
	Blo t 41	Blo t 41.0101	Putative chitin-binding protein (contains a peritrophin-A-like domain)	Respiratory	11,12
Bombyx mori (Silk moth)	Bomb m 3	Bomb m 3.0101	Tropomyosin	Food	13
	Bomb m 4	Bomb m 4.0101	30 kDa hemolypmh lipoprotein PBMHP-6	Food	14
	Bomb m 5	Bomb m 5.0101	30 kDa lipoprotein	Food	-
	Bomb m 6	Bomb m 6.0101	Hemolymph lipoprotein 3	Food	15
***Broussonetia papyrifera* (Paper mulberry)**	Bro p 3	Bro p 3.0101	Non-specific lipid transfer protein class 1 (nsLTP1)	Respiratory	16
***Callinectes bellicosus* (Warrior swimming brown crab)**	Cal b 2	Cal b 2.0101	Arginine kinase	Food	17
Cannabis sativa (Indian hemp)	Can s 2	Can s 2.0101	Profilin	Respiratory	18
	Can s 5	Can s 5.0101	Pathogenesis-related protein PR-10, Bet v 1-related protein	Respiratory	18
	Can s 7	Can s 7.0101	Thaumatin-like protein	Respiratory	19
Capsicum annuum (Chili, bell pepper)	Cap a 7	Cap a 7.0101	Gibberellin-regulated protein, snakin	Food	20
Charybdis feriata (Crucifix crab)	Cha f 10	Cha f 10.0101	Fructose bisphosphate aldolase	Food	-
Corylus avellana (Hazelnut)	Cor a 16	Cor a 16.0101<br>Cor a 16.0101 (29-714)<br>Cor a 16.0101 (715-1133)	7S globulin (vicilin) containing N-terminal alpha-hairpinin peptides	Food	21

Source	Allergen name	Isoallergens and variants	Biochemical names	Route of exposure	References
***Crassostrea angulate* (Portuguese oyster)**	Cra a 1	Cra a 1.0101	Tropomyosin	Food	22
	Cra a 2	Cra a 2.0101	Arginine kinase	Food	23
	Cra a 4	Cra a 4.0101	Sarcoplasmic calcium binding protein	Food	24
Cryptomeria japonica (Sugi, Japanese cedar)	Cry j 3 [a]	-	Thaumatin-like protein	Respiratory	25
Dermatophagoides farinae (American house dust mite)	Der f 40	Der f 40.0101	Thioredoxin like protein	Respiratory	26
	Der f 42	Der f 42.0101	Na/K-exchanging ATPase beta-subunit	Respiratory	27
	Der f 43	Der f 43.0101	Peroxiredoxin 1	Respiratory	27
	Der f 44	Der f 44.0101	Peroxiredoxin 2	Respiratory	27
Dermatophagoides microceras (House dust mite)	Der m 2	Der m 2.0101	NPC2 family; MD-2-related lipid recognition (ML) domain containing protein	Respiratory	28
Dermatophagoides pteronyssinus (European house dust mite)	Der p 16	Der p 16.0101	Gelsolin	Respiratory	-
	Der p 27	Der p 27.0101	Serpin serine protease inhibitor	Respiratory	-
	Der p 39	Der p 39.0101	Troponin C	Respiratory	29
	Der p 40	Der p 40.0101	Thioredoxin-like protein	Respiratory	-
Fagopyrum tataricum (Tartarian buckwheat)	Fag t 6	Fag t 6.01	Oleosin	Food	30
Helianthus annuus (Sunflower)	Hel a 15	Hel a 15.0101	2S albumin seed storage protein, SESA2-1	Food	31

Source	Allergen name	Isoallergens and variants	Biochemical names	Route of exposure	References
	Hel a 16	Hel a 16.0101	2S albumin seed storage protein, SESA20-2	Food	31
	Hel a 17	Hel a 17.0101	2S albumin seed storage protein, SFA-8 (SESA3)	Food	31
Humulus japonicas (Japanese hop)	Hum j 6	Hum j 6.0101	Pectin methylesterase inhibitor (pectinesterase inhibitor)	Respiratory	32
Juniperus oxycedrus (Prickly juniper)	Jun o 1	Jun o 1.01	Pectate lyase	Respiratory	-
***Lateolabrax maculatus* (spotted seabass)**	Late m 1	Late m 1.0101	Parvalbumin	Food	33
	Late m 2	Late m 2.0101	Parvalbumin	Food	33
Ligustrum vulgare (Common privet)	Lig v 2	Lig v 2.0101	Profilin	Respiratory	-
Litopenaeus vannamei (White shrimp)	Lit v 13	Lit v 13.0101	Cytosolic fatty acid binding protein	Food	34
Macrobrachium rosenbergii (Giant freshwater prawn)	Mac r 2	Mac r 2.0101	Arginine kinase	Food	-
***Mangifera indica* (Mango)**	Man i 1	Man i 1.0101	Class IV chitinase	Food	5
	Man i 2	Man i 2.0101	Pathogenesis-related protein PR-10; Bet v 1-related protein	Food	5
	Man i 4	Man i 4.0101<br>Man i 4.0102	Profilin	Food	5
Oryctolagus cuniculus (Rabbit)	Ory c 2	Ory c 2.0101	Odorant binding protein 2; lipocalin	Respiratory	35
***Panax ginseng* (Korean ginseng)**	Pana g 1	Pana g 1.0101	Pathogenesis-related protein PR-10-1, Bet v 1-related protein, ribonuclease 1	Food	-

Source	Allergen name	Isoallergens and variants	Biochemical names	Route of exposure	References
***Papaver somniferum* (Opium poppy)**	Pap s 1	Pap s 1.0101<br>Pap s 1.0101 (27-424)<br>Pap s 1.0101 (425-846)	Vicilin (7S globulin) containing N-terminal alpha-hairpinin peptides	Food	36
	Pap s 2	Pap s 2.0101<br>Pap s 2.0201	Legumin (11S globulin) seed storage protein	Food	36
	Pap s 3	Pap s 3.0101<br>Pap s 3.0201	Late embryogenesis abundant protein 5 (LEA-5); small hydrophilic seed protein	Food	36
***Paralithodes camtschaticus* (Red king crab)**	Para c 11	Para c 11.0101	Mitochondrial malate dehydrogenase	Food	37
Penaeus monodon (Black tiger shrimp)	Pen m 7	Pen m 7.01<br>Pen m 7.0101	Hemocyanin	Food	38
	Pen m 14	Pen m 14.0101	Glycogen phosphorylase-like protein	Food	38
Periplaneta americana (American cockroach)	Per a 4	Per a 4.0101<br>Per a 4.0102	Lipocalin-related calycin superfamily member	Respiratory	39,40
	Per a 8	Per a 8.0101	Myosin light chain	Respiratory	40
	Per a 14	Per a 14.01	Enolase	Respiratory	41
	Per a 15	Per a 15.01	Cytochrome c	Respiratory	41
	Per a 16	Per a 16.01	Cofilin	Respiratory	41
	Per a 17	Per a 17.01	Alpha-tubulin	Respiratory	41
	Per a 18	Per a 18.01	Peptidyl-prolyl cis-trans isomerase; cyclophilin	Respiratory	41
	Per a 19	Per a 19.01	Porin 3	Respiratory	41
	Per a 20	Per a 20.0101	Peroxiredoxin-6 (Prx6)	Respiratory	41

**Source**	**Allergen name**	**Isoallergens and variants**	**Biochemical names**	**Route of exposure**	**References**
Platanus acerifolia (Platanus x hispanica) (London plane tree)	Pla a 4	Pla a 4.0101	Profilin	Respiratory	42
	Pla a 5	Pla a 5.0101	Adenosylhomocysteinase (S-adenosyl-L-homocysteine hydrolase, AHCY)	Respiratory	-
	Pla a 6	Pla a 6.0101	Enolase	Respiratory	43
	Pla a 7	Pla a 7.0101	Triosephosphate isomerase (TIM)	Respiratory	44
	Pla a 8	Pla a 8.0101	Fructose-bisphosphate aldolase	Respiratory	-
***Portunus trituberculatus* (Gazami crab)**	Por t 4	Por t 4.0101	Sarcoplasmic Ca-binding protein (SCP)	Food	45
Prunus dulcis (Almond)	Pru du 1	Pru du 1.0101	Pathogenesis-related protein PR-10; Bet v 1-related protein	Food	46
Prunus persica (Peach)	Pru p 10	Pru p 10.0101	Polygalacturonase	Respiratory	-
***Quercus acutissima* (Sawtooth oak)**	Que ac 1	Que ac 1.0101	Pathogenesis-related protein PR-10; Bet v 1-related protein	Respiratory	47
	Que ac 2	Que ac 2.0101	Profilin	Respiratory	47
Scylla paramamosain (Mud crab)	Scy p 1	Scy p 1.0101	Tropomyosin	Food	48
	Scy p 3	Scy p 3.0101	Myosin light chain	Food	49
	Scy p 9	Scy p 9.0101	Filamin C	Food	50
***Solea solea* (Sole)**	Sole s 1	Sole s 1.0101	Parvalbumin	Food	-
***Trichiurus lepturus* (Atlantic cutlassfish, largehead hairtail)**	Tric l 1	Tric l 1.0101	Parvalbumin	Food	-
Tyrophagus putrescentiae (Storage mite)	Tyr p 4	Tyr p 4.0101	Alpha-amylase	Respiratory	-

Source	Allergen name	Isoallergens and variants	Biochemical names	Route of exposure	References
	Tyr p 11	Tyr p 11.0101	Paramyosin	Respiratory	51
	Tyr p 31	Tyr p 31.0101	Cofilin	Respiratory	52
	Tyr p 32	Tyr p 32.0101	Inorganic pyrophosphatase	Respiratory	53
***Zanthoxylum bungeanum* (Sichuan pepper)**	Zan b 1	Zan b 1.0101	2S albumin	Food	54
	Zan b 2	Zan b 2.0101<br>Zan b 1.0102<br>Zan b 1.02<br>Zan b 1.03	11S globulin (legumin) seed storage protein	Food	55

[a] For Cry j 3, no isoallergen designations were assigned, because the provided sequence information obtained from the purified natural allergen was not sufficient to unambiguously identify the IgE-binding isoforms expressed in *Cryptomeria* pollen.

Table S2. New isoallergens and variants of previously identified allergens approved by the WHO/IUIS Allergen Nomenclature Sub-Committee between 2021 and 2024.

Source	Allergen name	Isoallergens and variants	Biochemical names	Route of exposure	References
Ambrosia trifida (Giant ragweed)	Amb t 8	Amb t 8.0201	Profilin	Respiratory	-
Corylus avellana (Hazelnut)	Cor a 1	Cor a 1.0302<br>Cor a 1.0501<br>Cor a 1.0601<br>Cor a 1.0701<br>Cor a 1.0801	Pathogenesis-related protein PR-10; Bet v 1-related protein	Respiratory<br>Respiratory<br>Food<br>Food<br>Food	56
	Cor a 11	Cor a 11.0102	7S globulin (vicilin) seed storage protein	Food	21
Daucus carota (Carrot)	Dau c 1	Dau c 1.0501<br>Dau c 1.0601	Pathogenesis-related protein PR-10; Bet v 1-related protein	Food	57
Dermatophagoides farinae (American house dust mite)	Der f 23	Der f 23.0201	Peritrophin-A-like protein	Respiratory	58
Equus caballus (Domestic horse)	Equ c 1	Equ c 1.0102<br>Equ c 1.0201	Lipocalin	Respiratory	59
Juglans regia (Walnut)	Jug r 2	Jug r 2.0102<br>Jug r 2.0102 (27-367)<br>Jug r 2.0102 (368-789)	7S globulin (vicilin) containing N-terminal alpha-hairpinin peptides	Food	60
Macadamia integrifolia (Macadamia)	Mac i 1	Mac i 1.0101 (28-76)	Alpha-hairpinin	Food	61
Periplaneta americana (American cockroach)	Per a 7	Per a 7.02	Tropomyosin	Respiratory	41
Triticum aestivum (Wheat)	Tri a 20	Tri a 20.0201<br>Tri a 20.0301	Gamma-gliadin	Food	62

Source	Allergen name	Isoallergens and variants	Biochemical names	Route of exposure	References
	Tri a 21	Tri a 21.0201<br>Tri a 21.0301<br>Tri a 21.0401<br>Tri a 21.0501	Alpha/beta-gliadin	Food	62
	Tri a 36	Tri a 36.0201<br>Tri a 36.0301	Low molecular weight glutenin	Food	62
Tyrophagus putrescentiae (Storage mite)	Tyr p 20	Tyr p 20.0201	Arginine kinase	Respiratory	51

References

1. Arnoldi I, Villa M, Mancini G, et al. IgE response to Aed al 13 and Aed al 14 recombinant allergens from *Aedes albopictus* saliva in humans. *World Allergy Organ J.* 2023;16(11):100836.
2. Xu YF, Li K, Zhu LX, et al. Identification of Amb t 18 as a novel allergen from *Ambrosia trifida* pollen. *Asian Pac J Allergy Immunol.* 2025;doi:10.12932/AP-120425-2065.
3. Wangorsch A, Lidholm J, Mattsson LA, et al. Identification of a defensin as novel allergen in celery root: Api g 7 as a missing link in the diagnosis of celery allergy? *Allergy.* 2022;77(4):1294-1296.
4. Perez-Riverol A, Hideki Izuka Moraes G, Dos Santos-Pinto JRA, et al. An allergomic study reveals two novel venom allergens, phospholipase A1 and antigen 5, from the social wasp *Apoica pallens*. *Clin Exp Allergy.* 2025;55(3):250-252.
5. Zhao L, Xie H, Wang X, et al. Molecular characterization of allergens and component-resolved diagnosis of IgE-mediated mango fruit allergy. *Allergy.* 2023;78(6):1699-1703.
6. Song LB, Zhang L, Zhu Y, et al. Identification of fructose-bisphosphate aldolase as new pollen allergens. *Allergy.* 2024;79(5):1368-1372.
7. Cheng YL, Xu ZQ, Wang H, et al. Molecular and immunological characterization of two polcalcins as novel allergens of *Artemisia sieversiana* pollen. *Allergol Int.* 2023;72(2):347-350.
8. Yang DZ, Tang J, Cheng YL, et al. Identification and characterization of pectate lyase as a novel allergen in *Artemisia sieversiana* pollen. *Int Arch Allergy Immunol.* 2024;185(11):1019-1032.
9. Rick EM, Woolnough K, Richardson M, et al. Identification of allergens from *Aspergillus fumigatus* - Potential association with lung damage in asthma. *Allergy.* 2024;79(5):1208-1218.
10. Suzuki K, Nakamura M, Sato N, Futamura K, Matsunaga K, Yagami A. Nattokinase (Bac s 1), a subtilisin family serine protease, is a novel allergen contained in the traditional Japanese fermented food natto. *Allergol Int.* 2023;72(2):279-285.
11. Xiong Q, Liu X, Wan AT, et al. Genomic analysis reveals novel allergens of *Blomia tropicalis*. *Allergol Int.* 2024;73(2):340-344.
12. Luo W, Zhang J, Zheng X, et al. Identification of rBlo t 41 with a chitin-binding type-2 domain: A novel major allergen from *Blomia tropicalis*. *Int J Biol Macromol.* 2024;262(Pt 1):129972.
13. Jeong KY, Han IS, Lee JY, Park KH, Lee JH, Park JW. Role of tropomyosin in silkworm allergy. *Mol Med Rep.* 2017;15(5):3264-3270.
14. Jeong KY, Lee JS, Yuk JE, et al. Allergenic characterization of Bomb m 4, a 30-kDa *Bombyx mori* lipoprotein 6 from silkworm pupa. *Clin Exp Allergy.* 2022;52(7):888-897.
15. Yue W, Huang S, Lin S, et al. Purification, immunological identification, and characterization of the novel silkworm pupae allergen *Bombyx mori* lipoprotein 3 (Bomb m 6). *J Agric Food Chem.* 2023;71(36):13527-13534.
16. Jiang Z, Yin X, Chen Z, Liu S, Meng J, Xu A. Bro p 3, an nsLTP1: The first major allergen identified in *Broussonetia papyrifera* pollen. *Mol Immunol.* 2025;182:160-170.
17. Brassea-Estardante HA, Martinez-Cruz O, Cardenas-Lopez JL, Garcia-Orozco KD, Ochoa-Leyva A, Lopez-Zavala AA. Identification of arginine kinase as an allergen of

brown crab, *Callinectes bellicosus*, and in silico analysis of IgE-binding epitopes. *Mol Immunol.* 2022;143:147-156.
18. Ebo DG, Decuyper, II, Rihs HP, et al. IgE-binding and mast cell-activating capacity of the homologue of the major birch pollen allergen and profilin from *Cannabis sativa*. *J Allergy Clin Immunol Pract.* 2021;9(6):2509-2512.
19. Ebo DG, Rihs HP, Mertens CH, et al. Exploring the thaumatin-like protein (TLP) as a candidate cannabis allergen in North-Western Europe. *Allergy.* 2024;79(1):257-259.
20. Takei M, Nin C, Iizuka T, et al. *Capsicum* allergy: involvement of Cap a 7, a new clinically relevant gibberellin-regulated protein cross-reactive with Cry j 7, the gibberellin-regulated protein from Japanese cedar pollen. *Allergy Asthma Immunol Res.* 2022;14(3):328-338.
21. Mattsson L, Holmqvist M, Porsch H, et al. A new vicilin-like allergen in hazelnut giving rise to a spectrum of IgE-binding low-molecular-weight N-terminal fragments. *Clin Exp Allergy.* 2022;52(10):1208-1212.
22. Yun X, Li MS, Chen Y, et al. Characterization, epitope identification, and cross-reactivity analysis of tropomyosin: an important allergen of *Crassostrea angulata*. *J Agric Food Chem.* 2022;70(29):9201-9213.
23. Huan F, Han TJ, Liu M, et al. Identification and characterization of *Crassostrea angulata* arginine kinase, a novel allergen that causes cross-reactivity among shellfish. *Food Funct.* 2021;12(20):9866-9879.
24. Han TJ, Liu M, Huan F, et al. Identification and cross-reactivity analysis of sarcoplasmic-calcium-binding protein: a novel allergen in *Crassostrea angulata*. *J Agric Food Chem.* 2020;68(18):5221-5231.
25. Fujimura T, Futamura N, Midoro-Horiuti T, et al. Isolation and characterization of native Cry j 3 from Japanese cedar (*Cryptomeria japonica*) pollen. *Allergy.* 2007;62(5):547-553.
26. Cai ZL, Liu S, Li WY, et al. Identification of an immunodominant IgE epitope of Der f 40, a novel allergen of *Dermatophagoides farinae*. *World Allergy Organ J.* 2023;16(8):100804.
27. Cai ZL, Liu S, Ji A, Zhu R, Chen JJ, Ji K. Identification of three novel mite allergens, Der f 42, Der f 43, and Der f 44, from *Dermatophagoides farinae* by gelsolin interactome analysis. *World Allergy Organ J.* 2025;18(7):101067.
28. Hu RH, Wu CT, Wu TS, et al. Systematic Characterization of the Group 2 House Dust Mite Allergen in *Dermatophagoides microceras*. *Front Cell Infect Microbiol.* 2021;11:793559.
29. Li WY, Cai ZL, Zhang BP, Chen JJ, Ji K. Identification of an immunodominant IgE epitope of Der p 39, a novel allergen of *Dermatophagoides pteronyssinus*. *World Allergy Organ J.* 2022;15(5):100651.
30. Chen F, Li H, Fan X, et al. Identification of a novel major allergen in buckwheat seeds: Fag t 6. *J Agric Food Chem.* 2021;69(45):13315-13322.
31. Achour J, Reche M, Valbuena T, et al. Sunflower seed allergy: Identification of novel 2S-albumins as potential marker allergens. *Allergy.* 2024;79(8):2273-2276.
32. Jeong KY, Sang M, Lee YS, Gadermaier G, Ferreira F, Park JW. Characterization of Hum j 6, a major allergen from *Humulus japonicus* pollen, the primary cause of weed pollinosis in East Asia. *Allergy Asthma Immunol Res.* 2023;15(6):767-778.
33. Liu Q, Sui Z, Feng N, et al. Characterization, epitope confirmation, and cross-reactivity analysis of parvalbumin from *Lateolabrax maculatus* by multiomics technologies. *J Agric Food Chem.* 2024;72(36):20077-20090.

34. Munera M, Martinez D, Wortmann J, et al. Structural and allergenic properties of the fatty acid binding protein from shrimp *Litopenaeus vannamei*. *Allergy.* 2022;77(5):1534-1544.
35. Janssen-Weets B, Kerff F, Swiontek K, et al. Mammalian derived lipocalin and secretoglobin respiratory allergens strongly bind ligands with potentially immune modulating properties. *Front Allergy.* 2022;3:958711.
36. Podzhilkova A, Nagl C, Hummel K, et al. Poppy seed allergy: molecular diagnosis and cross-reactivity with tree nuts. *J Allergy Clin Immunol Pract.* 2024;12(8):2144-2154.
37. Li S, Bian J, Xiong Q, et al. Revealing the Diverse Allergenic Protein Repertoire of Six Widely Consumed Crab Species: A Species-Specific Allergen in King Crab. *Allergy.* 2025;doi:10.1111/all.16674.
38. Wai CYY, Leung NYH, Leung ASY, et al. Comprehending the allergen repertoire of shrimp for precision molecular diagnosis of shrimp allergy. *Allergy.* 2022;77(10):3041-3051.
39. Tan YW, Chan SL, Ong TC, et al. Structures of two major allergens, Bla g 4 and Per a 4, from cockroaches and their IgE binding epitopes. *J Biol Chem.* 2009;284(5):3148-3157.
40. Wangorsch A, Jamin A, Eichhorn S, et al. Component-resolved diagnosis of American cockroach (*Periplaneta americana*) allergy in patients from different geographical areas. *Front Allergy.* 2021;2:691627.
41. Wang L, Xiong Q, Saelim N, et al. Genome assembly and annotation of *Periplaneta americana* reveal a comprehensive cockroach allergen profile. *Allergy.* 2023;78(4):1088-1103.
42. Yang YS, Xu ZQ, Zhu W, et al. Molecular and immunochemical characterization of profilin as major allergen from *Platanus acerifolia* pollen. *Int Immunopharmacol.* 2022;106:108601.
43. Jiao YX, Song LB, Xu ZQ, et al. Purification and characterization of enolase as a novel allergen in *Platanus acerifolia* pollen. *Int Immunopharmacol.* 2022;113(Pt A):109313.
44. Song LB, Jiao YX, Xu ZQ, et al. Identification of Pla a 7 as a novel pollen allergen group in *Platanus acerifolia* pollen. *Int Immunopharmacol.* 2023;125(Pt A):111160.
45. Zhu W, Zhao J, Huang Y, et al. Purification, expression, and characterization of sarcoplasmic calcium binding protein: a novel allergen of *Portunus trituberculatus*. *J Agric Food Chem.* 2023;71(28):10773-10786.
46. Kabasser S, Crvenjak N, Schmalz S, et al. Pru du 1, the Bet v 1-homologue from almond, is a major allergen in patients with birch pollen associated almond allergy. *Clin Transl Allergy.* 2022;12(8):e12177.
47. Jeong KY, Lee J, Sang MK, et al. Sensitization profile to sawtooth oak component allergens and their clinical implications. *J Clin Lab Anal.* 2021;35(7):e23825.
48. Liu GY, Mei XJ, Hu MJ, et al. Analysis of the allergenic epitopes of tropomyosin from mud crab using phage display and site-directed mutagenesis. *J Agric Food Chem.* 2018;66(34):9127-9137.
49. Li MS, Xia F, Liu M, et al. Cloning, expression, and epitope identification of myosin light chain 1: an allergen in mud crab. *J Agric Food Chem.* 2019;67(37):10458-10469.
50. He XR, Cheng YM, Yang Y, et al. Cloning, expression and comparison of the properties of Scy p 9, a *Scylla paramamosain* allergen. *Food Funct.* 2020;11(4):3006-3019.

51. Zhou Y, Klimov PB, Gu X, et al. Chromosome-level genomic assembly and allergome inference reveal novel allergens in *Tyrophagus putrescentiae*. *Allergy.* 2023;78(6):1691-1695.
52. Zhou D, Ren Y, Zhou Y, et al. Expression, purification, and activity of novel allergen Tyr p 31 from *Tyrophagus putrescentiae*. *Int J Biol Macromol.* 2024;258(Pt 1):128856.
53. Zhou DM, Ren YN, Liao YF, Zhou Y, Cui YB. Production of recombinant protein of Tyr p 32 from *Tyrophagus putrescentiae* and identifying its immunoreactivity. *Zhonghua Yu Fang Yi Xue Za Zhi.* 2024;58(12):1933-1939.
54. Li H, Zhu L, Wang RQ, et al. A new pepper allergen Zan b 1.01 of 2S albumins: Identification, cloning, characterization, and cross-reactivity. *Asian Pac J Allergy Immunol.* 2024;doi:10.12932/AP-300423-1595.
55. Hu J, Zhu LP, Wang RQ, et al. Identification, characterization, cloning, and cross-reactivity of Zan b 2, a novel pepper allergen of 11S legumin. *J Agric Food Chem.* 2024;72(14):8189-8199.
56. Hendrich JM, Reuter A, Jacob TP, et al. Allergenicity and structural properties of new Cor a 1 isoallergens from hazel identified in different plant tissues. *Sci Rep.* 2024;14(1):5618.
57. Hendrich JM, Wangorsch A, Rodel K, Jacob T, Mahler V, Wohrl BM. Allergenicity and IgE Recognition of New Dau c 1 Allergens from Carrot. *Mol Nutr Food Res.* 2023;67(3):e2200421.
58. Yi MH, Yong TS, Kim CR, Jeong KY, Kim JY. IgE-sensitization and cross-reactivity of Der f 23 and Der p 23 in Korean patients with allergy. *Asian Pac J Allergy Immunol.* 2025;doi:10.12932/AP-120924-1924.
59. Janssen-Weets B, Lesur A, Dittmar G, et al. Proteomic analysis of horse hair extracts provides no evidence for the existence of a hypoallergenic Curly Horse breed. *Clin Transl Allergy.* 2024;14(2):e12329.
60. Lyons SA, Datema MR, Le TM, et al. Walnut allergy across Europe: distribution of allergen sensitization patterns and prediction of severity. *J Allergy Clin Immunol Pract.* 2021;9(1):225-235.
61. Kabasser S, Pratap K, Kamath S, et al. Identification of vicilin, legumin and antimicrobial peptide 2a as macadamia nut allergens. *Food Chem.* 2022;370:131028.
62. Aoki Y, Yagami A, Sakai T, et al. Alpha/beta gliadin MM1 is a novel antigen for wheat-dependent exercise-induced anaphylaxis. *Int Arch Allergy Immunol.* 2023;184(10):1022-1035.
